# The Recyclability of Fire-Retarded Biobased Polyamide 11 (PA11) Composites Reinforced with Basalt Fibers (BFs): The Influence of Reprocessing on Structure, Properties, and Fire Behavior

**DOI:** 10.3390/molecules29133233

**Published:** 2024-07-08

**Authors:** Mateusz Barczewski, Aleksander Hejna, Jacek Andrzejewski, Joanna Aniśko, Adam Piasecki, Adrian Mróz, Zaida Ortega, Daria Rutkowska, Kamila Sałasińska

**Affiliations:** 1 Institute of Materials Technology, Poznan University of Technology, Piotrowo 3, 61-138 Poznan, Poland; 2 Institute of Materials Engineering, Poznan University of Technology, Jana Pawła II 24, 60-965 Poznan, Poland; 3 Mechanical Engineering Institute, Collegium Mechanicum, The President Stanislaw Wojciechowski Calisia University, 4th Nowy Świat Street, 62-800 Kalisz, Poland; 4 Departamento de Ingeniería de Procesos, Campus Universitario de Tafira Baja, Universidad de Las Palmas de Gran Canaria, 35017 Las Palmas de Gran Canaria, Spain; 5 Faculty of Materials Science and Engineering, Warsaw University of Technology, Wołoska 141, 02-507 Warsaw, Poland

**Keywords:** polyamide, PA11, bio-polyamide, mechanical recycling, waste management, intumescent flame retardant, composite, basalt fibers, flammability, circular economy

## Abstract

The growing requirements regarding the safety of using polymers and their composites are related to the emergence of more effective, sustainable, and hazardous-limited fire retardants (FRs). Significant amounts of FRs are usually required to effectively affect a polymer’s burning behavior, while the knowledge of their recycling potential is still insufficient. At the same time, concerns are related not only to the reduced effectiveness of flame retardancy but also, above all, to the potential deterioration of mechanical properties caused by the degradation of temperature-affected additives under processing conditions. This study describes the impact of the four-time reprocessing of bio-based polyamide 11 (PA11) modified with an intumescent flame-retardant (IFR) system composed of ammonium polyphosphate (APP), melamine cyanurate (MC), and pentaerythritol (PER) and its composites containing additional short basalt fibers (BFs). Composites manufactured via twin-screw extrusion were subjected to four reprocessing cycles using injection molding. A comprehensive analysis of their structural, mechanical, and fire behavior changes in each cycle was conducted. The obtained results confirmed the safety of using the proposed fire-retarded polyamide and its composites while reprocessing under the recommended process parameters without the risk of significant changes in the structure. The partial increase in flammability of reprocessed PA-based materials caused mainly by polymer degradation has been described.

## 1. Introduction

One of the fundamental problems of polymer material recycling is the heterogeneity of polymers in the waste stream [1,2], including contamination with various difficult-to-separate polymer fractions with similar chemical structures but different thermal and processing properties, as well as the presence of unknown fillers and additives [3]. The increasing overall share of polymeric materials used as components of cars and rail vehicles and their presence in construction and civil engineering are constantly subjected to novel restrictions regarding flammability for safety reasons, which is associated with the introduction of flame retardants (FRs). Various mechanisms of their impact, including those based on thermal degradation and the simultaneous release of non-flammable gases, make it possible to degrade and deteriorate the structure subjected to recycling materials by improper processing.

Polyamide (PA), since Du Pont Corporation introduced polyamide under the trade name Nylon in 1928, has become the essential structural thermoplastic to form highly durable parts of machines intended for elevated-temperature working conditions [4]. Depending on the structure of the monomer, there are different varieties of polyamide, such as 6, 6.6, 4.6, 11, 12, or 1012 [4,5,6,7]. In its structure, polyamide 11 (PA11) contains 10 CH_2_ groups between the amide group in the monomer. Since this material contains a smaller amount of hydrogen bonds than the most common PA6 and PA66, it has lower water absorption properties and, therefore, is more resistant to water- and temperature-induced degradations at melt-processing conditions and better stability of properties compared to the two most common grades mentioned [7,8]. Moreover, PA11 is one of the varieties of polyamide that can be produced from renewable raw materials. For this reason, it is gaining more and more industrial interest, and further ways of modifying it are being sought to expand its scope of applicability. The literature describes the possibility of reducing the flammability of PA11 with various compounds, including [9] intumescent flame retardants such as ammonium polyphosphate (APP) [10] or modified inorganic nanofillers, such as phosphorus-modified halloysite [11]. This polymer shows excellent flame retardancy, as described by different testing methods and resulting from various mechanisms. All the considered works [9,10,11] showed a decrease in the temperature at the beginning of polyamide degradation after modification. An important question becomes how recycling processes can affect the potential limitations of the effectiveness of FRs.

Many efforts have been made in the existing literature to describe the changes in the structure of various thermoplastic polymers caused by repeated processing [12,13,14,15]. Changes in the properties of composites were also analyzed, including those reinforced with particle-shaped fillers or fibers [16,17] and plant-based fillers [18]. Interestingly, despite the high importance of application and industry needs, the number of studies concerning the changes in the structure of thermoplastic composites containing flame retardants, including changes in the effectiveness during burning, is limited [19]. Zhang and co-workers [20] investigated the influence of screw configuration in the extruder plastifying system on changes in the properties of polyamide 4.6 reinforced with glass fiber (GF) and containing decabromodiphenyl ethane (DBDPE) and Sb_2_O_3_ as flame retardants. However, no reprocessed PA in flammability increase was noted as part of the technological work carried out with composite processing temperature below DBDPE degradation temperature. Regardless of the configuration of mixing systems used in single- and twin-screw extruders, all compositions retained a comparable limited oxygen index (LOI) and V-0 flammability class despite the deterioration of mechanical properties correlating directly with applied shear intensity during processing. The work focused on identifying changes caused only by a single reprocessing. FR-containing polymer recycling is crucial because its market share is still growing. Moreover, the industry is concerned about activating the intumescent effect, which may cause porosity due to reprocessing.

On the other hand, Davis et al. [21] focused on the impact of adding an inorganic filler (montmorillonite clay, MMT) on the intensification of degradation phenomena occurring during the melt processing of PA. It was shown that the presence of the filler increased the intensity of the polymer thermal degradation phenomenon, probably resulting from catalytic activity, as well as an increased water absorption of the composite material. The residual moisture contained in the composite, which is more difficult to remove in preprocessing, causes additional degradation sources, including hydroxyls. On this basis, it can be concluded that the recycling processes of complex systems containing additives might increase the hydrophilicity of the polymer. Therefore, the study of ammonium polyphosphate (APP) [22,23,24] and inorganic fillers with confirmed water uptake [25,26] requires an in-depth analysis.

This study aimed to investigate multiple processing carried out using an injection molding process on changes in the properties of flame-retarded PA11 containing IFRs and short basalt reinforcing fibers (BFs) in its structure. The comprehensive analysis included determining mechanical properties under static and dynamic loads and thermomechanical properties correlated with changes in the material structure determined via scanning electron microscopy. These studies were related to the changes in burning behavior determined via cone calorimetry. The work is preliminary research and aims to describe potential threats or exclude uncontrolled side effects regarding the impact of processing on a selected series of polyamides with reduced flammability.

## 2. Results and Discussions

### 2.1. Structure Analysis

**Figure 1:**
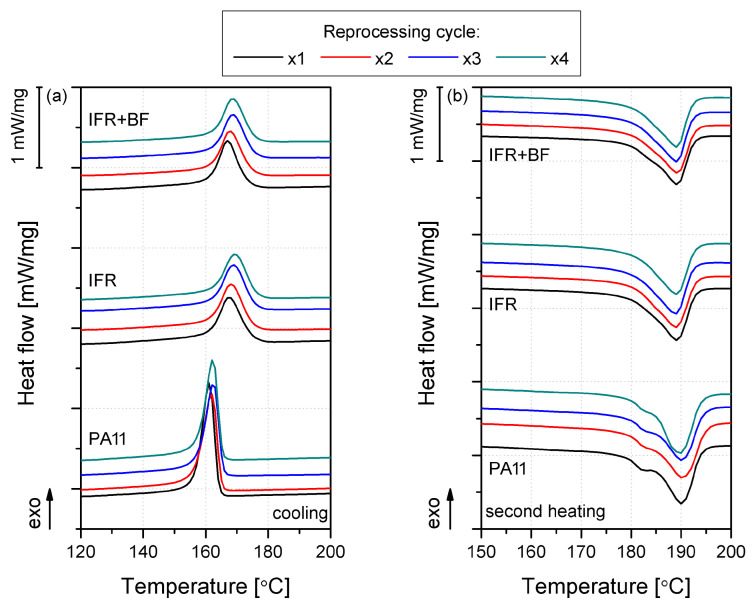
Cooling (**a**) and second heating (**b**); DSC thermograms measured for PA and its composites subjected to multiple reprocessing.

The thermal properties and changes in the crystallization process influenced by the addition of IFRs and BFs and the reprocessing were assessed using differential scanning calorimetry. The results in the form of DSC thermograms obtained during cooling and subsequent heating are presented in Figure 1, while detailed data on the characteristic temperatures, including crystallization temperature (T_C_), melting temperature (T_M_), and the degree of crystallinity (Xc) determined based on second melting, are collectively summarized in Table 1. The introduction of additives to PA11 increased T_C_, matching a higher crystallinity, indicating the nucleating effect of IFRs on the PA11 matrix. Previous work shows that APP, used in this study as a component of the IFR system, can act as a nucleating agent for various semicrystalline polymers [27]. The impact of BFs on different varieties of PA11 has been reported in earlier studies; for instance, Song et al. [28] showed a strong nucleating interaction of BFs in polyamide 1012, resulting in a 10 °C increase in T_C_. On the other hand, Patti et al. [29] showed a reduction in the crystallinity of polyamide 6.6 modified with basalt fibers. The effect of increased crystallinity in the case of IFR+BF composites was slightly lower than for the IFR series. Considering the fact of reducing the content of the flame retardant with a confirmed nucleating effect, it can be assumed that the BFs themselves did not additionally change the thermal properties of the composite by promoting the crystallization process. Although only a ~5 °C increase in the crystallization temperature was recorded, this change may be significant from the point of view of processing using high-performance methods, such as large parts injection molding, which may bring about the beneficial effects of shortening the technological cycle time [30], as well as a more uniform crystallinity in part volume [31]. A higher uniformity of properties will positively influence the mechanical properties of the final product, being an additional benefit of the proposed system. In the case of all material series, no significant shift in melting temperature values was recorded, placed at approximately 189 °C. Only for the unmodified PA11 series was a double peak observed in the heating cycle, with an additional inflection with a maximum of 182 °C. The double melting effect is due to crystal reorganization during the relatively slow heating used in the DSC test (10 °C/min) [7] and the crystallization of polyamide in at least two crystallographic forms [32,33]. In the case of other materials, the impact of the dispersed additional phase (IFRs and BFs), acting as heterogeneous nucleants, increased the number of active nucleation centers, which probably minimized this effect by promoting crystallization in a more thermodynamically stable form.

**Figure 2:**
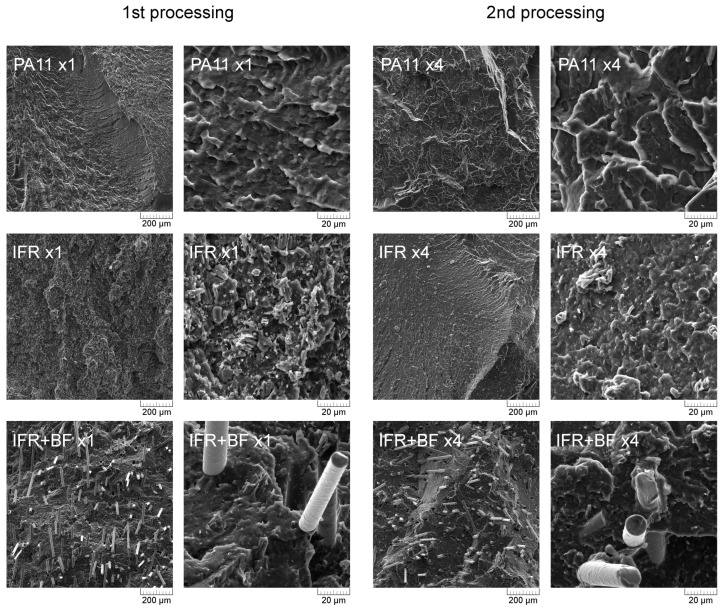
SEM images of brittle fractured injection-molded samples in two magnifications after 1st and 4th processing.

SEM images of brittle fractured cross-section structures of injection-molded samples taken in two magnifications after the first and fourth processing cycles are presented collectively in Figure 2. Both PA11 series (x1 and x4) show the features of a typical semicrystalline fracture with a developed fracture surface. The fracture surface observed at higher-magnification SEM images is much more complex for all series in the first processing cycle (marked with index x1), indicating plastic deformation dominated the fracture mechanism. The smoother surface of the reprocessed samples may be correlated with the increased crystallinity, as was discussed by Park et al. [35], and is correlated with a different toughness of the samples. Repeated processing resulted in the beneficial effect of homogenizing the structure of the dispersed flame retardants, which is especially visible in images taken at a higher magnification. After the fourth technological process, the IFR+BF series is characterized by a shortening of the fibers present in the matrix and an increased number of pull-out holes. Those effects may be related to the partial degradation of the silane coating on the surface of the basalt fibers [36,37] or the reduction in the interphase surface area resulting from the shortening of the fibers and, thus, with an increased susceptibility to pull-out during external loads, causing breakage [38]. However, considering the observations of the polymer–basalt fiber interfacial area in SEM images taken in higher magnification, it is possible to emphasize the lack of gaps and detachments of the fiber from the matrix, which shows proper interphase adhesion. What is most important in the discussed microstructure analysis, both for IFR and IFR+BF, is that reprocessing did not result in any noticeable presence of pores, which could be caused by the thermal decomposition of flame retardants or the degradation of the polyamide matrix itself.

**Figure 3:**
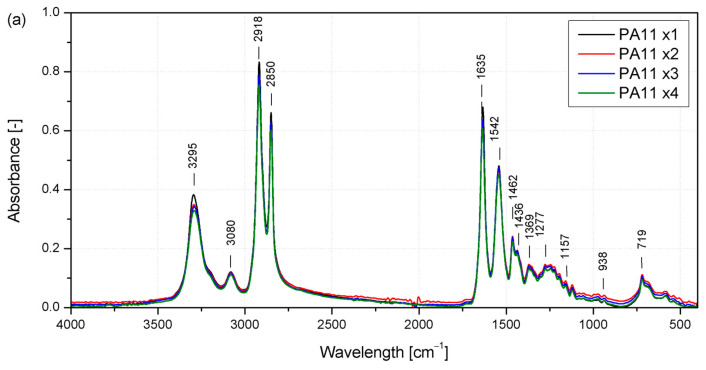

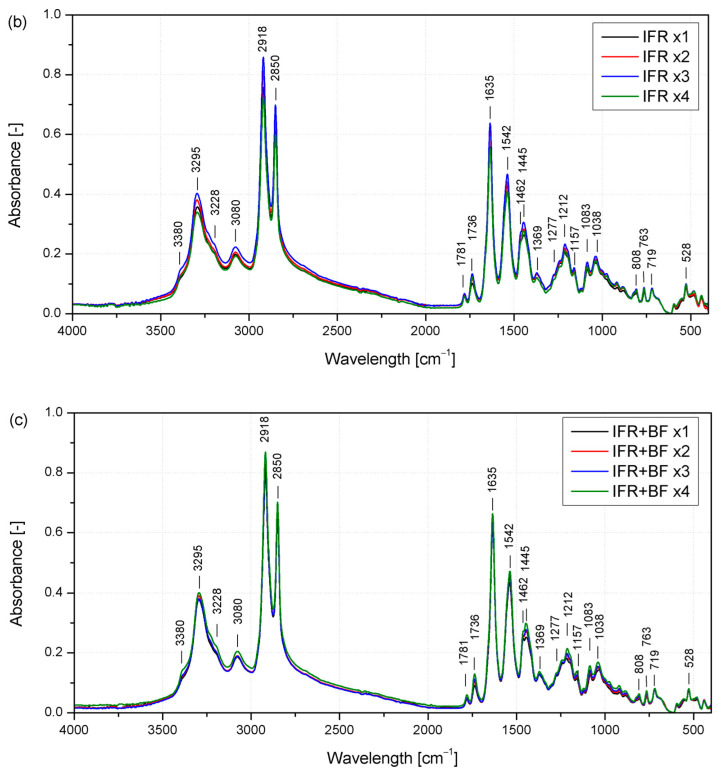
FTIR spectra of PA11 (**a**); IFR (**b**); and IFR+BF (**c**) after reprocessing.

Changes in the chemical structure of polyamide and the reprocessed polyamide composites were assessed using FTIR-ATR. In Figure 3, FTIR spectra of all material series are presented, and absorption bands characteristic of PA11 are observed, namely N-H stretching strong band (3295 cm^−1^), NH groups weak band (3080 cm^−1^), CH_2_ asymmetric stretching (2918 cm^−1^), CH_2_ symmetric stretching (2850 cm^−1^), Amide I, C=O stretching (1635 cm^−1^), Amide II, C-stretching and C=O in-plane bending (1542 cm^−1^), CH_2_ asymmetrical bending (1462 cm^−1^), N-H deformation (1436 cm^−1^), CH_2_ symmetrical bending (1369 cm^−1^), Amide III-NH-O stretching (1277 cm^−1^), interaction between NH bending deformation and O=C-N stretching (1157 cm^−1^), Amide IV-C-C(O) stretching mode (938 cm^−1^), and CH_2_ rocking and C=O deformation (719 cm^−1^) [39,40,41,42]. Based on the spectroscopic analysis, it can be assumed that no significant changes in the chemical structure of the polymer matrix occur in the case of unmodified PA11 due to the reprocessing of the material. The spectra of the consecutively processed polymer series showed no additional peaks or shifting.

According to Pliquet [40], two absorption bands in the case of polyamide were considered to describe degradation changes caused by thermo-oxidation phenomena, which may occur in this case because of repeated exposure to high temperatures by reprocessing in a molten state. The first is in the 3150–3000 cm^−1^ range, related to the amide II band, associated with N-H bond deformation and the stretching of the C-N bond in the amide group [40,43]. Its disappearance may be related to the degradation of polyamide. The spectra obtained for PA11 subjected to subsequent processing processes were characterized by decreased values after each cycle, but the changes can be considered negligible, as seen in Figure 3a. The second range is related to carbonyl group formation (1800–1700 cm^−1^). For unmodified PA11 in this range, two negligibly low-intensity absorption peaks can be noted at 1709 cm^−1^ and 1731 cm^−1^, attributed to isolated carboxylic acids and imides [40,44]. Based on the FTIR analysis, it can be concluded that the chemical structure of the polyamide itself did not undergo thermal and thermo-oxidative degradation under the processing and recycling process conditions used. Additional absorption bands in the curves of modified PA11 and the composite at 1781 cm^−1^, 1736 cm^−1^, 1445 cm^−1^, 1212 cm^−1^, 1083 cm^−1^, 1038 cm^−1^, 808 cm^−1^, 763 cm^−1^, 528 cm^−1^, and 438 cm^−1^ come from those reported in the literature for the flame retardants used, i.e., ammonium polyphosphate, melamine cyanurate, and pentaerythritol [45,46]. Additionally, two shoulders in the peak centered at about 3300 cm^−1^—one at 3380 and the other at about 3228 cm^−1^—can be found, attributed to the vibration of NH_4_^+^ in APP, which is also visible at 1440 cm^−1^. The slight changes observed at 1245 cm^−1^ and 1080 cm^−1^ are related to the phosphorous vibrations (P=O and PO_3_ in HPO_4_^−^, respectively) [22,45]. Finally, the increased intensity in the absorption found at about 1000 cm^−1^ is attributed to the C-OH vibrations in pentaerythritol [47]. No characteristic bands reported for basalt fibers were recorded in the case of composite samples originating from vibrations of metallic oxides (450 cm^−1^), as well as vibrations of Si-O and Al-O structures (780 cm^−1^) or the stretching of those bands at 1000 cm^−1^ [48]. Their absence may be explained by the surface nature of the measurements and the formation of a solid polymer coating during injection molding [49,50], in which the finely dispersed powder fillers were found near the surface of the shaped product, while the fibers oriented in the direction of flow were not close enough to the injection-molded sample wall layer. This effect can be enhanced by a small amount of BFs (10 wt%) in the volume of the tested sample.

### 2.2. Mechanical Properties

**Figure 4:**
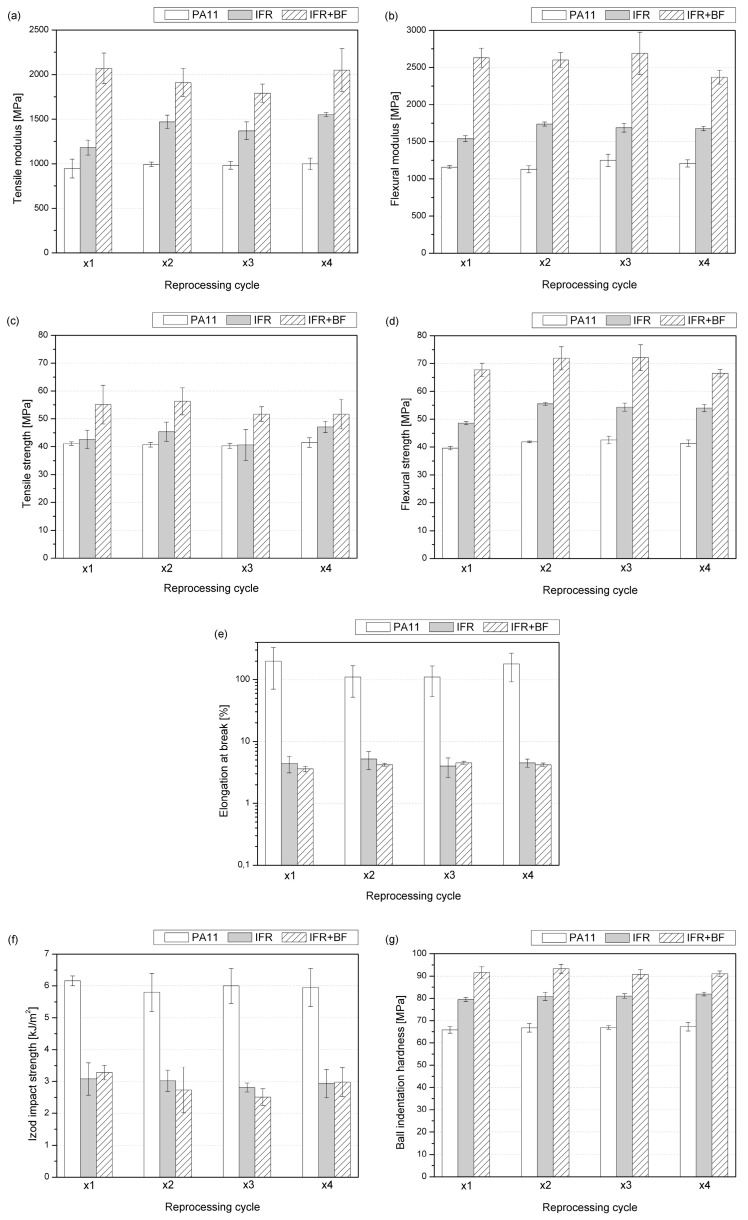
Mechanical properties of reprocessed polyamide, tensile (**a**,**c**,**e**), flexural (**b**,**d**), Izod impact (**f**), and ball indentation hardness (**g**) tests.

The mechanical properties of PA11 modified with IFRs and additionally reinforced with basalt fiber, subjected to four-time melt processing, are summarized in Figure 4. The comprehensive analysis included the determination of the tensile/flexural strength, the elastic modulus and elongation at break in the static tensile (a,c,e) and bending (b,d) tests, the determination of impact strength using the Izod method (f), and the ball indentation hardness (g).

Changes in PA11 stiffness caused by introducing the three-component flame-retardant system and short basalt fibers are comparable in the tests performed in tensile and bending modes. The addition of IFRs resulted in a 24% and 32% increase in elastic modulus determined in tensile and flexural tests compared to unmodified PA11, while the additional incorporation of BFs increased these values to 119% and 127%, respectively. Interestingly, subsequent processing cycles improved stiffness in the case of unmodified and IFR-modified polyamide, and the composites showed a gradual deterioration in these mechanical parameters. For PA11 and IFRs, the changes may be associated with changes in crystallization mechanism and the improved dispersion of an IFR acting as a nucleant [27,51]. The reduction in IFR+BF composites’ stiffness results from shortening inorganic fibers. This effect, caused by mechanical loads during melt processing and grinding, resulted in limited stress transfer efficiency. Analyzing the recorded values of elongation at break indicates no significant changes in the behavior of the materials. Regarding the most significant changes recorded for the PA11 series, the average values remain within the range of standard deviations. However, the IFR- and IFR+BF-modified systems showed a reduction in the elongation value typical of polymer composites containing more than 10 wt% of the filler and remain comparable to the literature data [52,53,54]. The decrease in elongation at break results from the interaction of dispersed modifier particles and fibers as disorganized particles in the polymer matrix and micrometric notches constituting a point of stress accumulation [55]. As a result, the stresses are not distributed uniformly throughout the sample, as with pure PA11. 

In most cases, introducing a micrometric size and an insoluble additive to thermoplastic polymers—constituting a separate phase—results in a deterioration of their impact strength compared to the unmodified material [56,57,58,59]. This results from accumulating stresses at the interfacial region during dynamic loading; particle-shaped fillers and additives dispersed in the polymer matrix can be treated as stress accumulation points and micrometric notches [60]. Therefore, the 50% reduction in Izod’s impact strength caused by the 30% addition of the flame-retardant system is understandable. Similar results were reported for other flame retardants chemically unreactive with polyamide [56]. Xu et al. showed that adding aluminum diethylphosphinate (AlPi) without a coupling agent or chain extender also caused a deterioration of the impact strength. The lower intensity of unfavorable changes than those observed in the considered case resulted from using at most 13 wt% of the fire retardant. The introduction of short BFs and the IFRs did not result in significant additional deterioration of the impact strength. Despite not treating the IFR series as a composite, both systems should be considered highly filled polymers. For the series marked IFR+BF, a similar trend was observed as for the flame-retardant one; the impact strength gradually decreased until the third processing, and in the fourth processing, it showed a value slightly lower than that of the material processed once. As reported by Gupta et al. [61], fiber shortening caused by subsequent processing cycles carried out using a single-screw plastifying unit takes place mainly in the melting zone at the solid–melt interface. The reported changes in fiber length occurred, especially for glass fibers that were longer than 8.0 mm. Medium-length fibers largely retained their dimensions, and the emerging fine fraction increased the filler dispersion in the matrix. In this case, four-fold processing was used via injection molding and not extrusion as in [61]; therefore, the length of the melting zone was much shorter, which probably meant that the changes in the mechanical properties of composites reinforced with basalt fibers were not that significant. Moreover, different resistances to process conditions cannot be ruled out between thin glass and basalt fibers in a polyamide, which will be verified in the future. 

The material series modified with IFRs and BFs characterized higher average hardness values than unmodified PA11. The addition of IFRs resulted in an increase in ball indentation hardness by 15 MPa and the additional introduction of inorganic fibers caused this increase to be by 27 MPa. Previously published works describe the influence of short basalt fibers or basalt derivatives on semicrystalline polymers [62], arriving at similar results. It results from the simultaneous nucleating effect of the fibers themselves and the presence of a rigid phase distributed in the volume of the composite. The material reprocessing did not decrease the hardness of any tested material series. An effect that may lead to a decrease in this mechanical parameter is the formation of porosity caused by the presence of hydrophilic fillers or the thermal decomposition of one of the components. However, none of the effects considered were observed in the SEM analysis. The study of the fracture structure of the tested materials excluded the significant formation of pores that could provoke a change in mechanical properties. Moreover, the DSC analysis proved that there was a lack of significant changes in crystallinity that accompanied subsequent processing. Considering the comparable impact on the hardness of different basalt derivatives reported in the literature [62], the lack of changes between the composite series caused by subsequent processing is also justified.

### 2.3. Thermomechanical Properties

**Figure 5:**
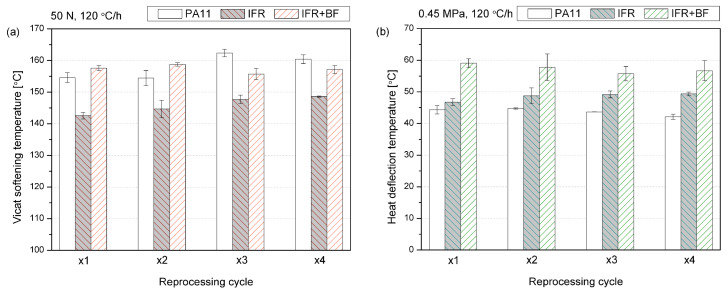
Thermomechanical properties of reprocessed polyamide; VST (**a**) and HDT (**b**).

From the application point of view, one of the most critical functional features required for polyamide is the stability of mechanical properties at elevated temperatures. Figure 5 summarizes the results of the thermomechanical analysis of the resistance of PA11 and its composites to point loads (VST) and in three-point bending (HDT). Comparing these two tests’ results is important due to the strong dependency on thermomechanical parameters obtained on the load type. Adding IFRs decreased the VST value compared to the sample made from neat PA11. This effect involves the dispersion of a low-hardness phase in the polymer matrix, with a simultaneous and probably insufficient change in the degree of PA11 crystallinity caused by the presence of IFRs, which could potentially counteract the reduction in VST. A similar effect of reducing VST caused by introducing 20 wt% of CaCO_3_, i.e., a filler with low hardness, into polypropylene resulted, despite an increase in the crystallinity observed and discussed in our former studies [63]. The additional presence of basalt fibers resulted in the suppression of adverse effects caused by flame retardants. Before reprocessing, the samples had a slightly higher VST compared to PA11. Subsequent reprocessing cycles increased VST, particularly for the PA11, with 6 °C increases in both cases compared to the first processed series. In the case of the composite series with basalt fibers, changes in VST do not show any visible trend and remain at a similar level.

The different relationship between the changes caused by the addition of the IFR system to PA11 in the VST and HDT results is due to the different reinforcing mechanisms analyzed in these two methods. VST results are dominated by changes in the crystalline structure and resistance caused by rigid filler inclusions located at the measuring point [64,65,66,67]. In the case of HDT, interactions at the phase boundary between the filler and polymer [68] aspect ratio and dispersion of the filler [69] play a much more significant role, which directly translates into an increase in the elastic modulus, the growth of which translates into an increase in the HDT value [69]. The presence of inorganic fibers with high stiffness resulted in a significant rise in HDT, consistent with the literature data obtained, among others, for a polypropylene composite reinforced with glass fibers [70]. Subsequent processing cycles increased HDT for the IFR series and slightly reduced it for the IFR+BF series. This may result from a more favorable dispersion of flame retardants in the polymer matrix caused by subsequent processing cycles and, in the second case, by the partial shortening of the inorganic fibers, causing a reduction in the effectiveness of their impact.

### 2.4. Fire Behavior

**Figure 6:**
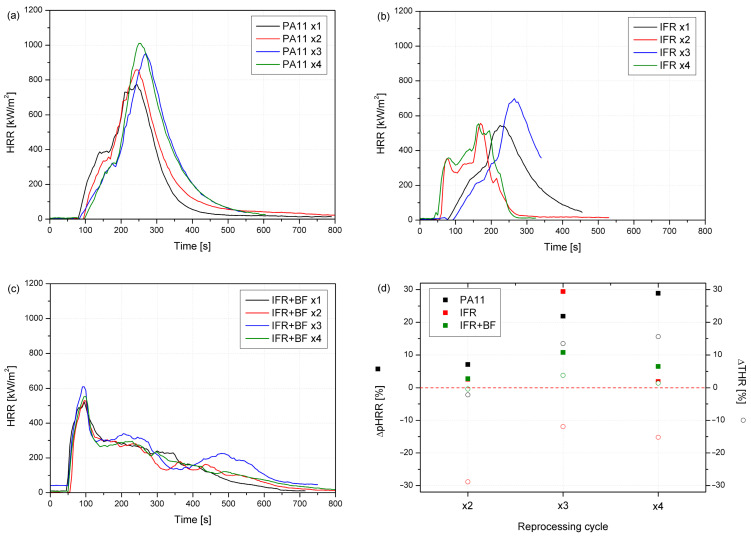
HRR vs. time curves (**a**–**c**) and relative changes in pHRR and THR caused by successive reprocessing cycles (**d**).

Burning behavior was assessed based on a cone calorimetry investigation. The average values of crucial parameters obtained from the tests are presented in Table 2, while Figure 6 shows representative curves of the heat release rates (HRRs) in the function of time or reprocessing cycle. PA11 showed two peaks, the second of which gave the maximum heat release rate (pHRR). Each subsequent processing resulted in increased heat release, related to PA11’s sensitivity to more elevated temperatures and the formation of shorter chains in the polymer. pHRR for PA11 x1 reached 774 kW/m^2^ and was the lowest of the polymers without additives. After applying four processing cycles, an increase in pHRR of approx. 30% was observed (Table 2). Employing a flame retardant reduced PA’s flammability and prevented it from increasing during subsequent cycles. The lowest pHRR values were observed for composites with basalt fibers and intumescent flame retardants. IFRs worked mainly in the condensed phase, while inorganic filler increased residue and reduced flammability by replacing the polymer with a less flammable component. The combination of IFRs and BFs reduced HRRs and flattened the curves, which is characteristic of samples that can form a char [71]. 

In the PA11 series, subsequent processing cycles reduced time to ignition (TTI), which was not observed for IFR-modified PA11 and its composites (Table 2). TTI values for the IFR series were lower, which might be due to the earlier decomposition of phosphorus flame retardant. The intensification of the decomposition processes as a result of the basalt application via increased thermal diffusivity was described in our previous work [72]. One of the important indicators determined on the basis of the HRR curves course is the maximum average rate of heat emission (MARHE). The MARHE may be used to predict full-scale fire development. The lowest MARHE was obtained for the IFR series, then composites, and the highest values for PA11, respectively. Reprocessing did not have such an impact on this parameter, as similar values without an unambiguous trend were obtained. The lowest total heat release (THR) values were observed for the IFR series, while the highest was for the PA one. This could be due to the burning time of the samples, which determined the duration of the test and the time of collecting measurement data. As can be seen in Figure 6, the test lasted from 300 to 500 s, while for the composites, it was about 800 s. The influence of burning time is particularly visible in the case of smoke emission analysis. The total smoke release (TSR) presented in Table 2 for the PA11 series with flame retardants is, in some cases, almost half as low. The reduction in THR may follow incomplete combustion from char forming, characteristic of intumescent flame retardants, the replacement of part of the polymer with less flammable components like basalt, or reduced combustion efficiency. Since the reduction in the effective heat of combustion (EHC) for IFR+BF compared to PA11 can be observed, none of the mechanisms can be excluded. In the case of EHC, no effect of the number of cycles on the parameter value was observed.

### 2.5. Thermal Stability and Analysis of Evolved Gaseous Products

The thermal stability analysis of the different materials processed was performed to determine whether the fillers or the reprocessing influenced their degradation characteristics. Table 3 summarizes the thermal parameters obtained from a thermogravimetric analysis (TGA), i.e., temperatures determined at characteristic mass loss values, 5, 10, and 50% (T_5%_, T_10%_, T_50%_); onset temperature (T_onset_) measured as the point of intersection of tangents adjusted to the course of the TG curve at the end of significant changes in its course; and the final results of these effects (T_final_). Moreover, detailed information about DTG peaks, the characteristic temperature and intensity of the degradation, as well as residue after reaching the maximum measurement temperature are presented.

**Figure 7:**
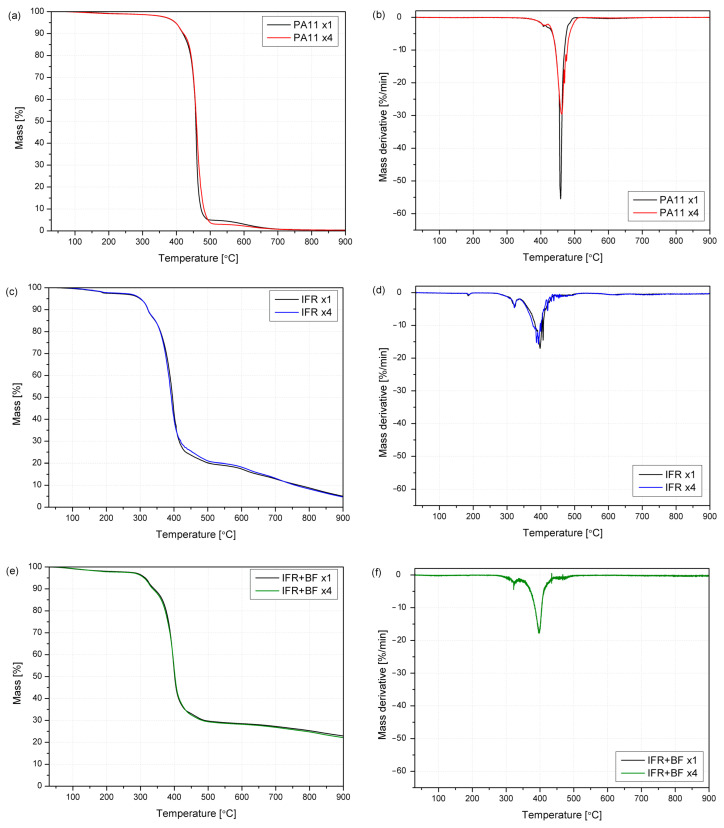
TG and DTG curves for PA11 (**a,b**), IFR (**c,d**), and IFR+BF (**e,f**) after the first and fourth processing.

It is observed that the behavior of the different series is not significantly affected by the reprocessing, as more clearly observed in Figure 7. The values of the degradation onset temperatures, understood as 5% mass losses, as well as T_onset_, do not show significant changes in each of the tested material series. The temperature values at 10 and 50% mass losses are also comparable, which proves the similar thermal stability of the reprocessed materials. However, some slight differences can be observed in derivative curves, where the peak of the maximum degradation rate is shifted to lower temperatures for modified PA11, taking the degradation place in a wider temperature range at a lower degradation rate (60.9%/min for PA11 x1 vs. 30.7%/min for PA11 x4). For the IFR-containing polyamide series, degradation occurs at lower temperatures, which is attributed to char formation and is characteristic of IFRs. Although employing IFRs caused a shift of peaks towards lower temperatures, the lower decomposition rates led to a higher residue yield. Improving the char formation is advantageous from a fire hazard point of view. 

Regarding derivative curves, the main degradation step for PA11 occurs at about 460 °C, with a minor peak at about 408 °C. The additional stages, corresponding to phosphoric acid dehydration and melamine sublimation, were found for the IFR series. During thermal decomposition, the melamine is partly volatilized, and cyanuric acid catalyzes the chain scission of the polyamides [73]. It is well known that adding pentaerythritol to APP provides a synergistic effect, although the ratio of both components needs to be optimized for each polymer matrix [74]. For instance, Xia et al. have found a ratio of 2:1 as optimum for a polypropylene matrix, based not only on studies about thermal stability and fire behavior but also on the analysis of the char formed. It can also be observed that adding basalt fibers plays a role in stabilizing the polymeric matrix, with an increase in the degradation temperatures and a reduction in mass loss.

**Figure 8:**
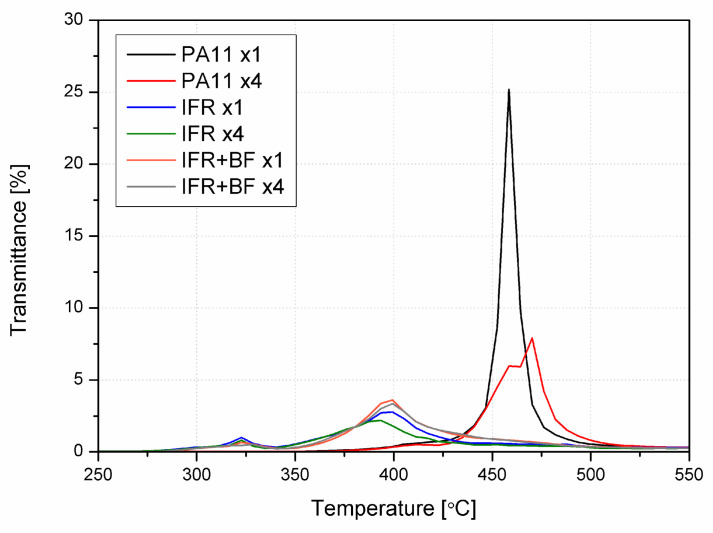
The Gram–Schmidt profile obtained from an analysis of the gases emitted during the TGA of PA11, IFR, and IFR+BF after the first and fourth reprocessing.

During the thermal analysis, exhaust gases were continuously analyzed via infrared spectroscopy. Figure 8 shows the Gram–Schmidt profile for the different samples. It was found that only one peak appeared for unmodified PA11, corresponding to the degradation of PA11 at about 460 °C. For the remaining samples, it is appreciated that there are mainly two stages, coinciding with the two steps of the degradation, one at about 320 °C (for the sublimation of melamine) and another one at about 400 °C, corresponding to the major mass loss. As the diagram for materials does not change due to reprocessing, only the curves for the first processing are shown. The achieved transmittance for neat PA11 is much higher than for the materials with IFRs, and as observed from TGA, the degradation takes place at higher temperatures. However, the matrix’s degradation occurs at a lower rate for those materials (IFR, IFR+BF) in a lower but broader range of temperatures, thus explaining the lower transmittance achieved for those samples. Moreover, this effect may be related to the lower content of PA in the modified material series. 

**Figure 9:**
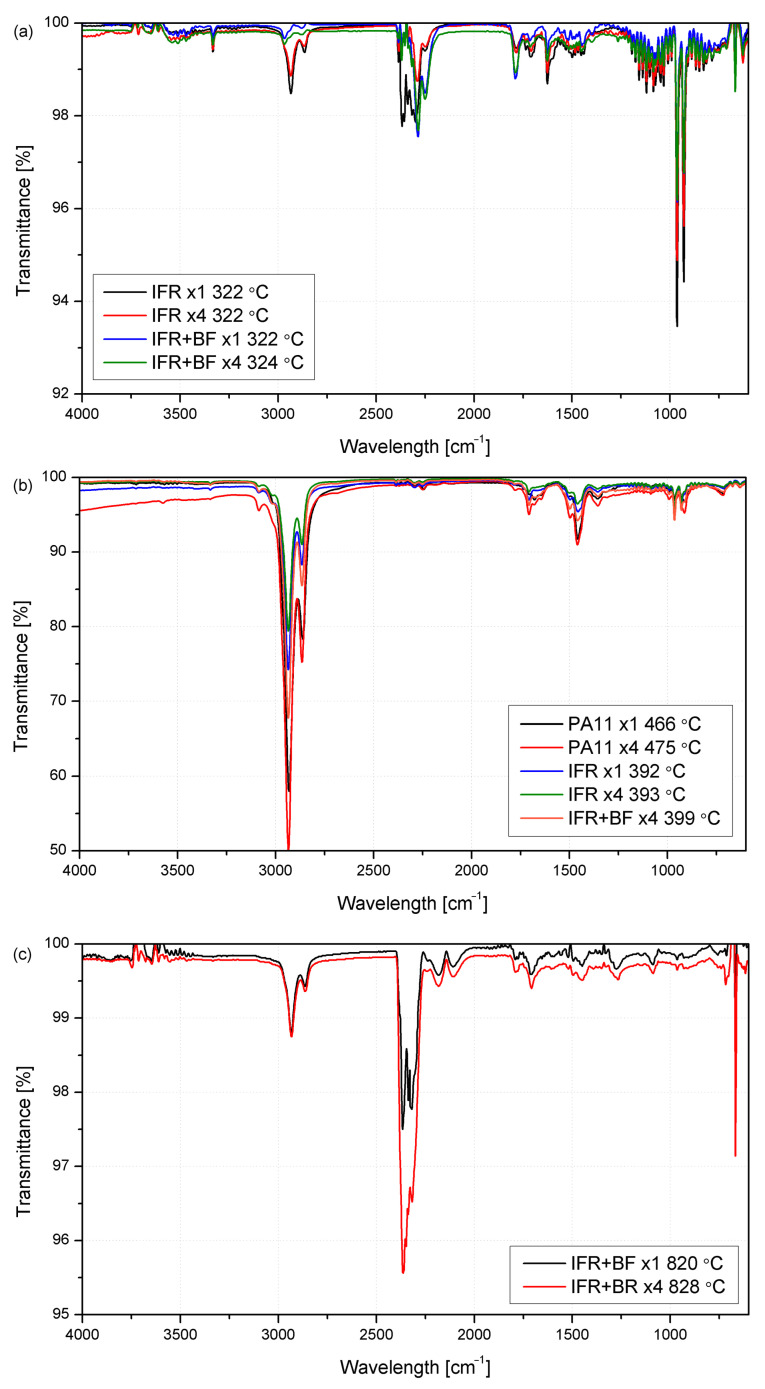
FTIR spectra of gases for each set of materials at the temperatures at which the most significant thermal events were recorded.

Figure 9a shows the spectra obtained for the first stage of degradation (only for those materials with IFRs, as nothing is visible from the PA11 series). The main bands found for these series of materials are related to the ammonia (NH_3_) release from APP (double peak at 930–965 cm^−1^ and bands from 1520 to 1670 cm^−1^), while the emission of CO_2_ is also found in the bands between 2300 and 2400 cm^−1^ [22]. As observed, the absorption bands for materials with basalt fibers are much weaker, particularly in the area of 2800–3000 cm^−1^ and mainly related to the C-H vibrations from pentaerythritol [46]. The reduced release of CO_2_ found in composites with basalt fibers or in the reprocessed modified PA11 (i.e., IFR x4 series) is the result of replacing organic material with a mineral component. As found in Figure 9b, despite the main degradation occurring at a reduced temperature for the materials with IFRs, the degradation path is not varied apparently, and degradation products are independent of the final composition of the blends. There are some weak bands under 1000 cm^−1^, related to the NH_3_ emission, coming from the decomposition of the polyamide; the bands at about 1700 cm^−1^ are attributed to C=O stretching in ketones, while the ones at 1500 cm^−1^ can be related to C=C-C in aromatic groups [75]. The most significant degradation products are found to be slightly under 3000 cm^−1^, related to C-H in CH_3_ and =CH_2_ stretching in alkenes, which are the main products of PA11 degradation [76]. Interestingly, the composites with basalt fiber show some release of CO_2_ at the latter stages (about 830 °C); these bands are not seen at this temperature for any other series of materials (Figure 9c).

## 3. Experimental Section

### 3.1. Materials, Sample Preparation, and Reprocessing Procedure

Polyamide 11 (PA11), Rilsan BMN O TLD NATURAL, with a density of 1.03 g/cm^3^, and the MVR = 30 cm^3^/10 min, was used as a polymeric matrix. The flame-retardant system consisted of three components: ammonium polyphosphate (APP) Addforce FR APP 201, melamine cyanurate (MC) Addforce FR MC 8, and pentaerythritol (PER) Addforce FR Penta M40, provided by WTH Walter Thieme Handel GmbH (Stade, Germany). The APP:MC:PER ratio in the system was 3:1:1. As an inorganic reinforcing fiber, chopped short basalt fibers (BFs) of type BCS 13-1/4″-KV02M with a length of 6 mm and diameter of 13 μm from Kamenny Vek company (Dubna, Russia) were used. Three series of materials were produced and analyzed, labeled in brackets: unmodified polyamide 11 (PA11), polyamide compositions with 30 wt% of the intumescent fire-retardant system (IFR), and a system containing 20 wt% IFRs and 10 wt% BFs (IFR+BF).

After weighing the components in batches, a physical mixing process was realized using Retsch GM 200 (3000 rpm, 3 min) (Retsch GmbH, Haan, Germany). Then, premixes were mixed in a molten state using a Zamak EH 16.D co-rotating twin-screw extruder (Zamak, Skawina, Poland) (100 rpm, 230 °C). Extrudates were cooled via forced airflow and pelletized. Before further processing, the materials were each dried using a Chemland vacuum dryer (Chemland, Stargard, Poland) (60 °C, 12 h). The 100 × 100 × 4 mm samples were manufactured via injection molding using the Engel Victory 50 machine (Engel GmbH, Schwertberg, Austria). The machine was equipped with a 25 mm screw and 500 kN clamping unit. The injection temperature was set to 230 °C (at the nozzle), and the mold temperature was 50 °C. The injection/holding pressure was 80/40 MPa, and the holding/cooling time was 10/40 s. The appropriate number of samples for mechanical, thermomechanical, structural, and flammability tests were manufactured. As part of the reprocessing procedure, the remaining injection molded specimens were ground using a Shini SC-1411 low-speed mill (Shini Plastics Technologies, Dongguan, China), and the obtained grindings were subjected to further re-molding via injection molding, using the same parameters as those described. Before testing, the specimens were conditioned for at least 72 h at room temperature and 30% relative humidity.

### 3.2. Methods

Differential scanning calorimetry (DSC) was applied to determine the changes in thermal behavior and indirectly assess the crystalline structure of polyamide and its composites. For the experiment, 5.0 ± 0.2 mg specimens were used. They were subjected to double heating/cooling from −50 to 240 °C (heating/cooling rate of 10 °C/min) under nitrogen flow. The measurements were performed in a Netzsch 204 F1 Phoenix apparatus (Netzsch-Gerätebau GmbH, Selb, Germany) and aluminum crucibles with pierced lids.

The scanning electron microscope (SEM) Tescan MIRA3 (Brno, Czech Republic) was used to perform the structure analysis of the PA11 and its composites. The evaluated samples were assessed with an accelerating voltage of 12 kV and a working distance of 16 mm. A thin carbon coating approximately 20 nm thick was deposited on samples using the Jeol JEE 4B vacuum evaporator (Jeol Ltd., Tokio, Japan). 

The Fourier-transform infrared spectroscopy (FTIR) measurements were carried out using a spectrometer Jasco FT/IR-4600 (Jasco Corporation, Tokyo, Japan) at room temperature (23 °C) in a mode of Attenuated Total Reflectance (ATR-FTIR). Sixty-four scans at a resolution of 4 cm^−1^ were used in all cases to record the spectra.

The mechanical properties of unmodified and modified PA11 were examined in the static tensile test according to ISO 527 standard [77] at a 50 mm/min crosshead speed. Flexural properties were measured according to ISO 178 [78] at 10 mm/min bending speed, with 64 mm between cantilevers, thereby determining the elastic modulus and flexural strength. The measurements were performed using a Zwick Roell Z010 universal testing machine (Zwick GmbH & Co. KG, Ulm, Germany) with a 10 kN nominal force gauge. At least 7 replicates per test for each series were conducted.

Izod impact resistance measurements were performed on the notched samples where the notch depth was 2 mm, according to ISO 180 [79]. The Zwick/Roell HIT 15 machine (Zwick GmbH & Co. KG, Ulm, Germany), with a hammer of a 5 J energy pendulum, was used. The test results presented are the average of at least 11 measurements.

The hardness evaluation was conducted using a KB Prüftechnik apparatus (KB Prüftechnik GmbH, Hochdorf-Assenheim, Germany) with a ball indentation hardness test, according to ISO 2039 standard [80]. A minimum of 15 measurements were taken for each material series.

The Vicat softening temperature (VST) and heat deflection temperature (HDT) were determined with a Testlab RV300C apparatus (Testlab, Warszawa, Poland). Measurements were performed in an oil bath following the ISO 306 and ISO 75 standards [81,82]. The VST measurement was conducted at a 120 °C/h heating rate and a 50 N load. The HDT B-type experiment was prepared with a heating rate of 120 °C/h and a load of 0.45 MPa.

Burning behavior was assessed using cone calorimeter tests conducted on the Fire Testing Technology apparatus (Fire Testing Technology Ltd., East Grinstead, Great Britain), following the ISO 5660-1 procedures [83]. The horizontally oriented cuboid specimens, with dimensions of 100 × 100 × 4 mm, were irradiated at a heat flux of 50 kW/m^2^. Spark ignition was used to ignite the pyrolysis products. An optical system with a silicon photodiode and a helium–neon laser provided a continuous survey of smoke. 

The thermal stability of samples was analyzed in a PerkinElmer TGA 4000 thermogravimetric analyzer (PerkinElmer Inc., Waltham, MA, USA) under an air atmosphere of 10 mL/min. A nominal of 15 mg samples was prepared in alumina crucibles and heated at 10 °C/min from 30 to 950 °C. The exhausted gases were further analyzed via FTIR in a PerkinElmer Spectrum 2 spectrophotometer (PerkinElmer Inc., Waltham, MA, USA). The spectra were collected along the entire heating cycle. The transfer line was set at 270 °C, and the gas flow in the measurement cell was 70 mL/min.

## 4. Conclusions

The research carried out and the impact of repeated processing on the structure and properties of polyamide 11 and its composites with basalt fiber, containing intumescent flame retardants, allowed for the verification of the questions asked. The implementation of the multiple melt processing in the temperature range up to 230 °C, and not exceeding the analytically determined ranges of the thermal decomposition onset of flame retardants, allowed for the conclusion that it is possible to safely use flame-retardant varieties of polyamide without the risk of producing defective products. 

The crystal structure of PA11 did not change due to extensive exposure to thermal and thermomechanical loading conditions, which may suggest the occurrence of significant degradation effects in the structure of the polymer and composites series. The mechanical and thermomechanical properties of both IFR and IFR+BF series were preserved, and the confirmed lack of significant amounts of porosity confirmed that the observed differences due to remanufacturing were not due to the initiation of IFR action. 

The conducted research on combined thermogravimetric and TGA-FTIR spectroscopic analysis and flammability assessment led to conclusions that the mechanisms of interaction of flame retardants and inorganic fibers, despite some variations in measured values, did not change their character. Therefore, it can be stated that there are no significant process-related degradative changes in the structure of modifiers. To sum up, this study contributes to supplementing knowledge in the field and confirming the possibility of processing fire-retarded thermoplastic polymers and their composites. The obtained tests allow us to confirm this group of materials’ recyclability and their high effectiveness and ability to be reused in industrial practice.

## Figures and Tables

**Table 1:** Thermal parameters of reprocessed PA11 and its composites.

Material	PA11				IFR				IFR+BF			
Processing cycle	1	2	3	4	1	2	3	4	1	2	3	4
T_C_ [°C]	161.1	161.8	162.3	162.5	167.5	168.1	168.8	169.5	166.9	167.8	168.6	169.7
T_M2_ [°C]	190.0 182.1	190.2 182.4	190.1 182.6	189.7 182.4	189.1	189.0	188.9	189.1	189.2	189.2	189.1	189.2
Xc * [%]	21.9	23.1	21.4	20.5	26.3	26.2	26.1	26.1	24.9	25.4	25.7	25.5

* Xc = ΔH_M2_/(ΔH_M100%_*(1 − Φ)), ΔH_M2_—melting enthalpy; ΔH_M100%_ for PA11 = 200 J/g [34], Φ—mass fraction of the filler.

**Table 2:** Cone calorimeter results of PA11, IFR, and IFR+BF.

Material	TTI	pHRR	MARHE	THR	EHC	TSR
	s	kW/m^2^	kW/m^2^	MJ/m^2^	MJ/kg	m^2^/m^2^
PA11 x1	76	774	351	89	36	1247
PA11 x2	74	829	327	130	29	1228
PA11 x3	65	944	354	127	35	1443
PA11 x4	70	998	362	147	37	1346
IFR x1	46	529	224	150	20	669
IFR x2	67	543	234	79	15	676
IFR x3	67	685	232	56	25	676
IFR x4	57	539	284	70	18	846
IFR+BF x1	58	507	261	67	28	726
IFR+BF x2	74	521	244	108	25	1709
IFR+BF x3	58	562	245	112	28	1565
IFR+BF x4	73	540	245	109	28	1699

**Table 3:** Summary of results from TGA.

Material	T_5%_	T_10%_	T_50%_	T_onset_		T_final_	DTG Peaks				Residue
	[°C]						[°C; %/min]				[%]
PA11 x1	397	419	457	447	-	466	-	-	407; −4.6	458; −60.9	0.4
PA11 x4	397	421	459	441	-	475	-	-	407; −3.2	460; −30.7	0.3
IFR x1	300	323	395	309	353	412	184; −0.2	322; −5.7	406; −43.7	-	3.5
IFR x4	301	323	392	306	346	408	186; −0.7	321; −10.7	392; −23.2	-	3.3
IFR BF x1	314	340	401	310	366	418	-	322; −4.4	397; −17.8	467; −1.5	21.8
IFR+BF x4	310	336	402	297	354	415	-	325; −2.6	399; −17.6	464; −1.2	21.0

## Data Availability

The data that support the findings of this study are available from the corresponding author upon reasonable request.
